# Using student-staff partnership to teach early years medical students about quality improvement: an evaluation

**DOI:** 10.1186/s12909-025-06779-7

**Published:** 2025-02-18

**Authors:** Cate Goldwater Breheny, Eve O’Connell, Lisa-Jayne Edwards, Noreen Ryan

**Affiliations:** 1 Imperial College School of Medicine, London, UK; 2 University of Warwick Medical School, Coventry, UK

**Keywords:** Medical education, Quality improvement, Qualitative research

## Abstract

**Background:**

Quality Improvement (QI) skills are recognized as a key outcome for medical students but are still rarely taught at the undergraduate level. Whilst there is evidence that preclinical students enjoy learning about QI, there is limited practical work exploring the best way to teach QI to this cohort. There are gaps in the literature around the evaluation of student-staff partnership approaches in the context of teaching QI, especially in line with the sustainable QI (susQI) framework. Our study evaluates a worksheet-based interactive session developed through student-staff partnership. The session was delivered to year one medical students in January 2024 at Imperial College School of Medicine (ICSM).

**Methods:**

An inductive approach to thematic analysis was used to review worksheet content submitted by students during the teaching session. This method was employed to determine the session’s effectiveness and ascertain common themes of interest and application. 16 groups comprising 69 total students submitted worksheets for analysis.

**Results:**

Three themes were identified: Making QI personal: learners chose to focus on improving their university experience over clinical projects, which affected how they identified stakeholders and designed their QI interventions; Misinterpretation of technical language: learners used their everyday understanding of QI terminology in the worksheets, evidencing some misconceptions; Understanding QI as a process: learners reported understanding and engaging with QI as a process and set of skills. Overall, students demonstrated both their understanding and application of QI methodology through engagement with the worksheet-based tasks.

**Conclusions:**

Student-staff partnership is a feasible tool to develop and deliver an engaging and effective teaching session on QI, in line with susQI principles, for students with limited clinical experience. Further evaluation and use of partnership approaches in developing QI teaching and curricula are welcome and will likely deliver positive outcomes for learners.

**Supplementary Information:**

The online version contains supplementary material available at 10.1186/s12909-025-06779-7.

## Background

### Introduction

Quality Improvement (QI) is recognised as a key outcome for medical graduates by the United Kingdom’s (UK) General Medical Council (GMC) and the World Health Organisation (WHO), with calls for medical students to have greater and earlier exposure to QI [1–3]. However, medical students today still have limited QI teaching, especially in preclinical years [4], despite increasing evidence that early QI education is feasible and well-received [5–8]. This may be because teaching is constrained by the limited evidence around how to best teach QI to early years medical students specifically: in particular, gaps exist around intervention design and duration [9]. To our knowledge, there are also no interventions described in the literature using student-staff partnership approaches in QI education for medical students.

Student-staff partnership approaches to education design involve students and staff working as equitable team members to develop and reflect on teaching [10]. These approaches have become increasingly embedded in UK higher education institutions, with evidence of increased student satisfaction and successful curriculum development [11, 12]. This has particular relevance for early years QI teaching, where students may struggle to engage due to their understandably limited experience in the clinical setting and packed didactic curricula [8]. Drawing on students’ expertise as partners allows teaching to meet students at their level of understanding, increasing engagement [11, 12].

At the same time, there is a growing recognition that medical education needs to do more to highlight sustainability and environmental health [13, 14] and this is often strongly supported by students [7]. QI education has been identified as a key opportunity to include this teaching for students using the Centre for Sustainable Healthcare’s Sustainability in Quality Improvement (SusQI) framework [7, 15]. This framework aims to use QI to foster healthcare that respects finite economic and environmental resources and adds positive social value to communities [16, 17]. However, it is only mentioned in two undergraduate educational interventions in the literature to date [7, 15], neither of which were developed in partnership with students. This offers further space for innovation in QI education in exploring how including susQI frameworks might be included and taught in sessions developed through student-staff partnership.

Our intervention sought to address these gaps through the design and delivery of a susQI-aligned teaching session for first year medical students through Imperial StudentShapers, a student-staff partnership scheme (18).

### Developing the session

This session was developed through a five-week paid StudentShapers Imperial College School of Medicine (ICSM) project. Authors CGB and EO’C were then year 3 undergraduate medical students who worked with the Professional Values and Behaviours (PVB) team [18]. PVB is an integrated module across the ICSM curriculum exploring medical ethics and law, quality in healthcare, and professional identity formation and behaviour. NR is Imperial’s PVB Domain Lead for Quality Healthcare and decided to target year 1 QI teaching for redevelopment through student partnership, as sessions had previously received poor student engagement. LJE was at the time a teaching fellow with the PVB team who further contributed to session brainstorming and design. At the time of writing, NR is a GP, LJE is a GP trainee, and CGB and EO’C are year five (senior) medical students.

To redevelop the session, CGB and EO’C reflected on teaching they had previously found engaging and their own experiences of peer teaching. They proposed ideas to restructure the session to NR and LJE. We all decided to build an interactive session beginning with a refresher quiz and including a guided worksheet taking students through the process of developing their own QI project, which they then ‘pitched’ to tutors and other groups at the end of the session. CGB and EO’C produced draft worksheets and session plans, which we developed iteratively through feedback and guidance from NR and LJE. Throughout the process, we aimed to work as equals to maintain the spirit of student-staff partnership [10] and position students as experts, giving CGB and EO’C the opportunity to lead on session conceptualisation and development.

### Context

Students at Imperial College School of Medicine (ICSM) take part in several QI learning activities across their first two years as a foundation for two summative QI projects in year three and for future QI work. A 90-minute interactive small-group session on QI was developed for first year students with limited clinical experience. We created a structured worksheet guiding students through a set of group tasks (an example blank worksheet can be seen in Appendix 1):

1. identifying a problem of their choosing (clinical or non-clinical);
2. identifying stakeholders and completing a power/interest stakeholder matrix;
3. exploring factors contributing to the problem;
4. considering potential solutions;
5. identifying their intervention’s social, economic, or environmental impacts following the SusQI framework.

Alongside the worksheet, learners would have access to a guide on an online learning platform and tutors would be available to facilitate discussion.

### Purpose of the study

This study aims to evaluate the educational value of an interactive QI teaching session developed through student-staff partnership. To determine students’ conceptual understanding of and engagement with QI concepts, group worksheets were collected and analysed.

## Methods

Ethical approval was granted (EERP2324-028a) to analyse anonymized student worksheets. All first-year students at ICSM (approx. 360) were timetabled to attend the session as a compulsory part of their curriculum with worksheets being used to facilitate a series of group tasks to apply QI concepts to an area of students’ choosing. Students were informed at the start of the session that they could opt-in to having their worksheets used in research through an online consent form. Students were asked to complete the online consent form independently of other group members and record the number of group members present on their worksheet. EO’C then collected all worksheets at the end of the session and compared them to the consent forms: where all members of the group had completed a valid consent form, worksheets were scanned for inclusion in data analysis and then transcribed by EO’C and CGB. All worksheets were then securely shredded. Students were thus selected through convenience sampling.

We chose to analyse student worksheets to explore students’ application of QI concepts in more depth. By analysing materials produced in the session, students did not have to do any additional work within the session. This increased engagement with research compared to requesting a lengthy feedback survey or interview. This method further reduced reliance on student self-reporting of learning and gave a measure of student competency, valuable in increasingly outcome-based QI curricula [4]. Including an optional reflective question around key learning points at the end of the worksheet al.so enabled us to explore changes in learner attitudes from the session, a facet of learning historically relatively underexplored in evaluations of QI teaching [4].

EO’C, CGB and NR performed an inductive thematic analysis of the worksheets [19]. EO’C and CGB read through the worksheets to develop initial codes, which were then refined in discussion with NR, resolving initial disagreements and queries around coding. EO’C and CGB then returned to the data and completed coding. All researchers (EO’C, CGB, NR and LJE) met to discuss and consolidate the themes and descriptors. This ensured we had agreed on coding and resolved issues by bringing our final codes together as a group. Results were synthesised into a written report: CGB and EO’C prepared the first draft and NR and LJE gave editorial assistance on the manuscript and all subsequent drafts.

## Results

The session ran in January 2024. 16 small groups (comprising 69 first year students in total) submitted anonymised worksheets for analysis. 9 worksheets were fully completed and 7 partially completed. The 7 partially completed worksheets did not have responses to the final question around students’ take-home messages from the session, possibly as this question was optional or because they did not have time to finish the full worksheet. No worksheets were missing responses to other questions. A blank worksheet is attached as Appendix 1. Example completed and partially completed worksheets can be seen in Appendix 2 and 3, respectively.

Three themes were derived from inductive data analysis of worksheet content:

1. Making QI personal.
2. Misinterpretation of technical language.
3. Understanding QI as a process.

### Theme 1: making QI personal

Students often selected ideas for QI projects that held personal resonance for them: 10 out of 16 groups focused on their university and teaching experience (Table 1). Student groups generated slightly more ideas for clinical projects (14 compared to 13 for university-based projects) but were less likely to choose to follow these through on the worksheet (Table 1).

**Table 1:** Student-generated topics for QI projects, which we have analysed as university-based or clinical placement-based. Note that some students proposed multiple projects, even if they ultimately selected one to focus on for the rest of the worksheet. We have highlighted the topics groups selected to focus on in bold.

University-based personal projects (13 total, 10 selected)	Clinical placement-based projects (14 total, 6 selected)
“More breaks in… sessions” (W2) **“£1 per session [attendance] or food vouchers [for attending the session]” (W2)** **““Unnable [sic] to find online… lectures to download [powerpoint slides] on [online learning platform]” (W7)** **“ No charging ports or plugs in [lecture halls]” W8)** “lack of accessibility to online educational materials” (W8) **“Navigating [online] learning platform: Navigating [online learning platform] difficult for medical students**,** no search bar.” (W9)** **“How to make teachers keep time better?” (W11)** **“lack of feedback on work submitted at university” (W35)** “Long lectures mean medical students are often in the same lecture theatre for up to three hours” (W35) **“Cleanliness of communal kitchens in student accommodation” (W49)** **“lack of attendance for small group sessions” (W53)** **“Attendance to placement– too far away” (W55)** **“to improve attendance by making…. sessions more convenient to attend” (W57)**	“Difficulty hearing patients on phone” (W2) “Posters for phone translators code” (W2) “Introduce video calls instead of phone calls [sic]”(W2) **“Patients don’t want to take medications due to inconvenience etc. but afraid of admitting to doctor” (W3)** **“how can we make the UK more inclusive to diabetics?” (W10)** **“Patients with English as not their first language may find it difficult to book appointments and access services**,** online.” (W12)** **“Helping disable [sic] and/or old people accessing the GP services independantly [sic]” (W13)** **“Inadequate translation services for non-emergency F2F GP appointments” (W21)** **“Our QI project addresses the disjuncture between primary care social care and secondary care that often results in inefficient care for patients due to poor communication” (W24)** **“**Patients who do not speak English as their first language often find it difficult to communicate across phone consultations” (W35) “Making communication with deaf patients more accessible” (W35) “Patients not understanding what the role of the GP is and as a result calling in the GP for tasks like renewing their prescription, that could have been done by the receptionist” (W35) “Lack of communication between different health care services meant there was sometimes confusion regarding patient’s latest test results for example” (W35) “patients going to the wrong care providers for their problems e.g A&E patients coming to GP, minor ailments coming to A&E” (W55)

All 10 learner groups who selected university-based personal projects identified medical students as stakeholders. Where medical students were identified as stakeholders in university-based project settings, they were almost always shown as having high interest (*n* = 8) and often as having moderate power (*n* = 7). However, medical students were only identified as stakeholders by half of the six learner groups who chose clinical placement-based projects. They were typically listed as having low interest (*n* = 2) and low power (*n* = 2).

Learners often proposed non-specific stakeholders for clinical projects, such as “*[the] NHS*” (Worksheet 13 [henceforth W13]) or “*government*” (W24).

For university-based projects, groups often identified more specific stakeholders such as “*academic tutors*” (W35, W55 & W57) or “*[hall] wardens*” (W35), but also proposed some non-specific stakeholders such as again “*the government*” (W53 & 57).

Students who selected university-based projects often proposed more detailed, multi-step interventions: “*As students*,* bring up to Academic Reps under student union -> SU -> Academic /technical team + academic tutor!*” (W7).

In comparison, learners who suggested clinical projects proposed less detailed interventions such as:

“*create a digital service with all patient information*” (W24).

### Theme 2: misinterpretation of technical language

When engaging with the worksheet, students often applied layperson understanding of the QI terminology used in the session. For example, learners often interpreted “social sustainability” as focussed on increasing participants’ network of relationships and sense of well-being, rather than social impact. Learners explained their projects would be “socially sustainable” because:

*“more students coming to lectures -> expands social circle”* (W8).

**Fig. 1:**
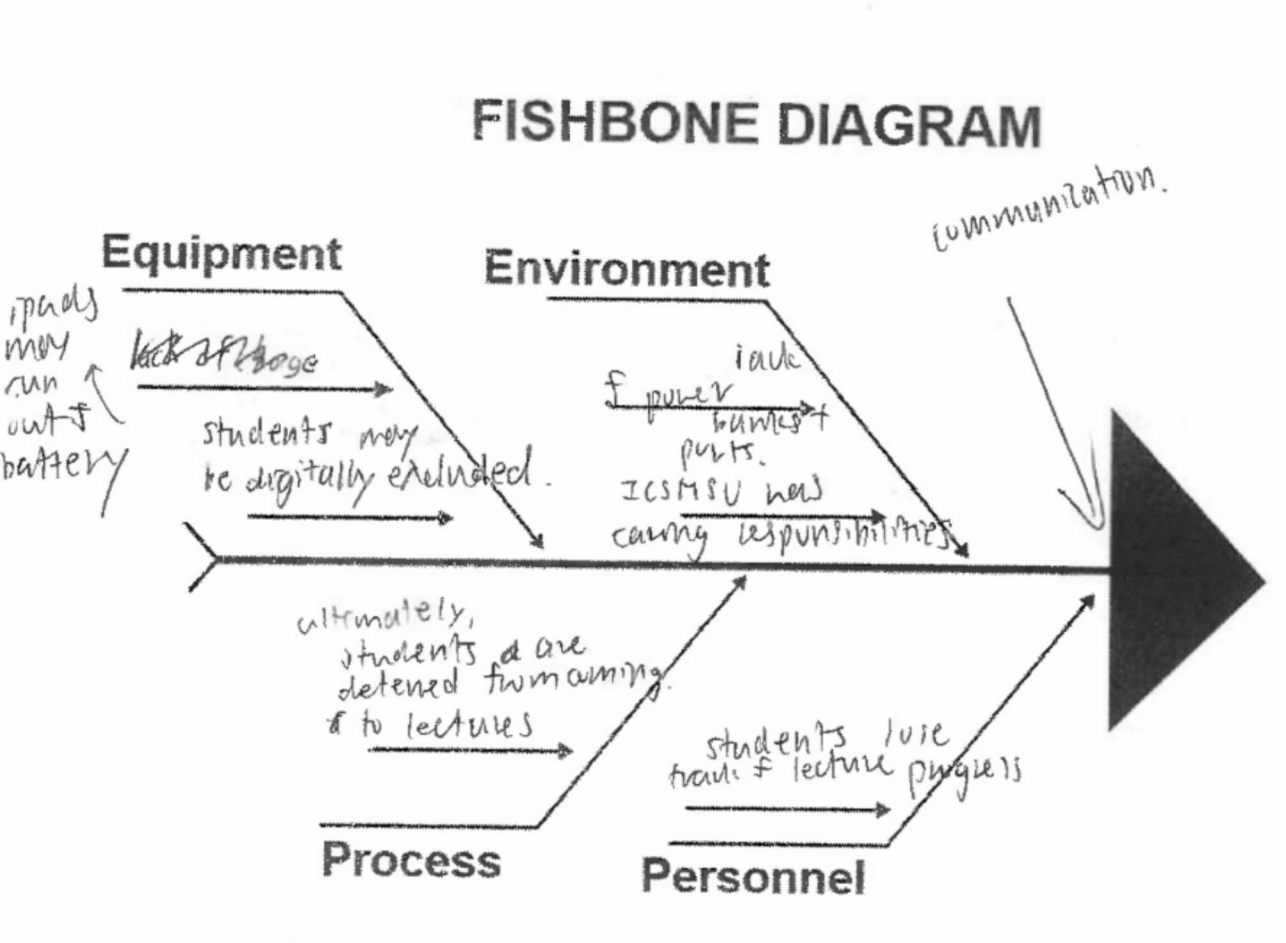
Fishbone diagram from worksheet 8, focusing on a lack of plugs in lecture halls, showing the group’s equation of process with the outcome of not solving the problem and a diversity of social and physical ideas under ‘environment’.

**Fig. 2:**
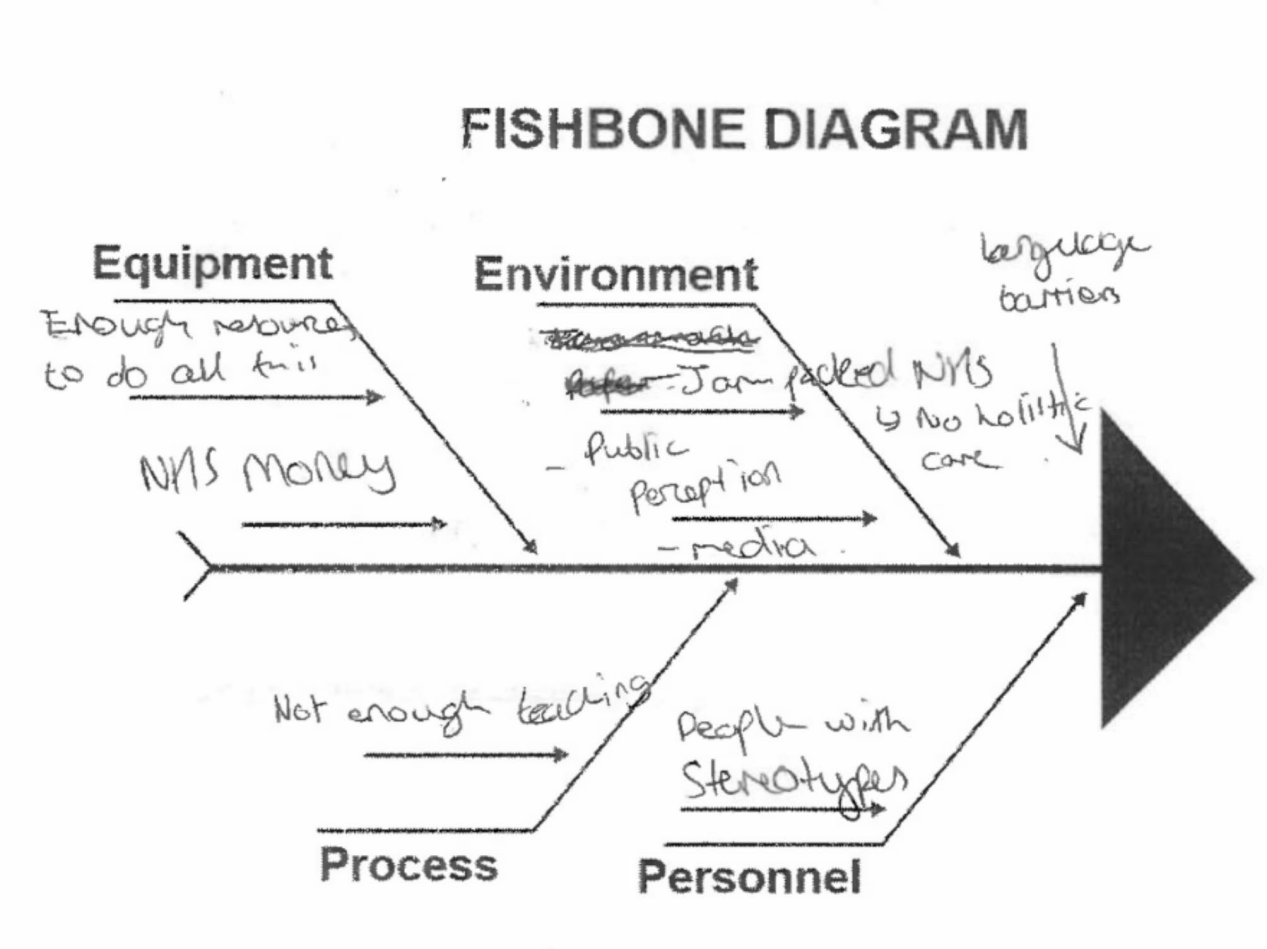
Fishbone diagram from worksheet 10, focused on improving healthcare experiences for people living with diabetes, showing social and personnel factors listed under ‘environment’.

Learners also interpreted the fishbone diagram (a cause analysis tool) labels using layperson understanding, with labels understood in different ways by different groups (see Figs. 1 and 2). In particular, students often seemed to conflate physical and social understandings of ‘environment’, effectively using it as a catch-all category.

### Theme 3: understanding QI as a process

Learners reported understanding and engaging with the process of QI, as scaffolded through the worksheet. They discussed their learning in terms of recognising QI as a complex process. However, their suggested interventions had limited scope, in contrast with students’ stated learning.

Students were asked at the end of the worksheet to summarise their take ‘take home messages’ from the session. These often highlighted their learning around using process-driven tools in QI and breaking down the problem:

*“[we learnt] use of fishbone diagram in planning”* (W2).

*“[we learnt to] break down the problem into different issues + categories to make it easier to address + find solutions for”* (W9).

However, learners’ proposed interventions instead often indicated a broad, rather than tailored, approach:

*“redesign booking system* [for GP appointments]*””* (W21).

“*harsher enforcement of rules* [around not attending teaching]” (W53).

## Discussion

This study adds to a growing body of evidence that educating early years medical students in QI is feasible [5–8]. Furthermore, it demonstrates the feasibility of student-staff partnership work in QI teaching.

While many prior studies of QI teaching activities use student surveys as their main form of data collection [9], this project analysed the output from the interactive worksheet-based task. We thus were able to evaluate engagement and learning in a simulated self-selected QI project directly and assess student competencies. Exploring student submissions also provides a valuable opportunity for educators in assessing the effectiveness of teaching. Examining the worksheets allowed faculty to see terms around SusQI had not been adequately scaffolded. As a result, further sessions have been adapted to make terminology clearer and to increase tutor support in these areas. This work further highlights a misalignment between student-reported learning and evidence of learning from tasks completed, which may be present in previous work exploring students’ self-reported learning.

Learners’ ability to plan a QI project in a classroom environment is likely limited by their inability to engage in meaningful information gathering [8], as evidenced by the broad solutions learners often proposed. Students’ lack of clinical experience may have informed the specificity and feasibility of proposed clinical projects, with the opposite being true for a setting where they were more familiar (i.e. university-based projects). Equally, it may also be that students struggle to understand systems that they are not directly exposed to, whether that is clinical practice or university governance, as they often proposed non-specific stakeholders in areas of education they did not directly engage with. However, this simulated, paper-based session may give early-years learners a foundation to apply to future QI projects: they often reported a new understanding of the complexities and challenges of the QI process.

Future study could explore further whether early exposure to an interactive QI session evoked long-term change in students’ attitudes to or engagement in QI projects in future. ICSM’s integrated QI teaching across the first three years of their medical degree may offer an interesting opportunity to track this. Further teaching giving students the opportunity to engage in real, on the ground QI projects [e.g. 20], while complex, may work well for this once core skills have been established: our work suggests that the educational setting offers key opportunities for students in QI.

This study represents an initial and promising analysis of the value of student-staff partnership in teaching early years medical students about QI, including susQI. There is scope to expand and research both student-staff partnership in medical education more broadly and QI more specifically. Exploring medical student perceptions of partnership-designed sessions can explore whether they may have use in other areas of the curriculum with poor engagement. Other work might look at developing a full QI curriculum through student-staff partnership or its applications in the postgraduate or clinical years setting.

## Conclusion

To our knowledge, this is the first analysis of a QI session developed through student staff partnership for early years medical students. We demonstrate that student-staff partnership enabled us to develop, deliver, and evaluate an engaging and effective teaching session on QI, including susQI, for students with limited clinical experience. Students demonstrated a foundational understanding of QI concepts and an appreciation of the complexities of the QI process that can be built upon later in their training. Our evidence suggests that student-staff partnership is a valuable tool for QI medical education and would benefit from further exploration and analysis.

### Author positionality

EOC and CGB are medical students at Imperial College School of Medicine. NR is a GP and the Domain Lead for Quality Healthcare at Imperial College School of Medicine and responsible for centralised QI teaching. LJE is a GP Specialist Trainee and was a Teaching Fellow in Professional Values and Behaviours at the time of the study.

The research team worked together as part of a StudentShapers [18] project during the Summer 2023. Throughout this project, the team have been aware of the challenge of existing staff-student hierarchies in student-staff partnerships, which are especially pronounced in the medical profession [21]. The team have stressed the importance of equality and open dialogue in our work together. All members of the team have been embedded the project from design to delivery and evaluation.

## Electronic supplementary material

Below is the link to the electronic supplementary material.

Supplementary Material 1

Supplementary Material 2

Supplementary Material 3

## Data Availability

The datasets generated and/or analysed during the current study are not publicly available to protect the privacy of the students who participated, but are available from the corresponding author on reasonable request.

## Abbreviations

GMC: General Medical Council
ICSM: Imperial College School of Medicine
NHS: National Health Service
QI: Quality Improvement
susQI: Sustainability in Quality Improvement
UK: United Kingdom
