# Supplementary material for: Using student-staff partnership to teach early years medical students about quality improvement: an evaluation
Source: BMC Med Educ. 2025 Feb 18;25:263. doi: 10.1186/s12909-025-06779-7 (PMC11837700; doi:10.1186/s12909-025-06779-7)
Supplement: Supplementary file 1 — Supplementary Material 1 [file 12909_2025_6779_MOESM1_ESM.pdf]

Group eg A5a:

Number of people in your group:

Thank you for considering PVB Quality Improvement for your QI funding needs! We want to support YOU to achieve your NHS quality boosting goals.

Before we can fund your project, we need to know more about the project you have planned. This easy form should take no more than the hour allocated in your session. To help you do this you have supportive materials on Insendi.

At the end of the session please place your form on the lead tutors' table if you are happy for it to be used as part of the student research project. You will also need to fill in a consent form via Qualtrics. This has been explained at the start of the session and on Insendi.

Well done for submitting your proposal to improve the quality of the service. We appreciate the thought you have put into the proposal and will give it proper consideration!

Worksheet Number:

Number of people in your group:

Task 1 - What problem does your QI project address?<br><br>Sell us with a one sentence summary.<br><br>This should take you around **10 minutes.**	
Task 2.1: Who does this problem matter to?<br><br>That is, who are your stakeholders?<br><br>Complete the stakeholder analysis diagram.<br><br>Bonus points: tell us how you would contact them (consider other means than surveys)!<br><br>This should take you around **15 minutes.**	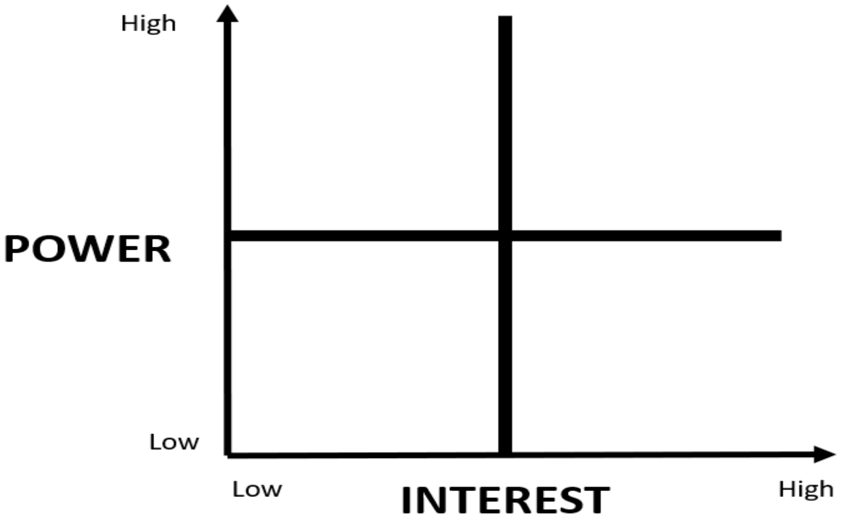

Task 2.2 - What makes the problem, a problem?<br><br>(Hint: Complete the fishbone diagram (aim to put at least two reasons per branch or add your own branches!))<br><br>This should take you around **15 minutes.**	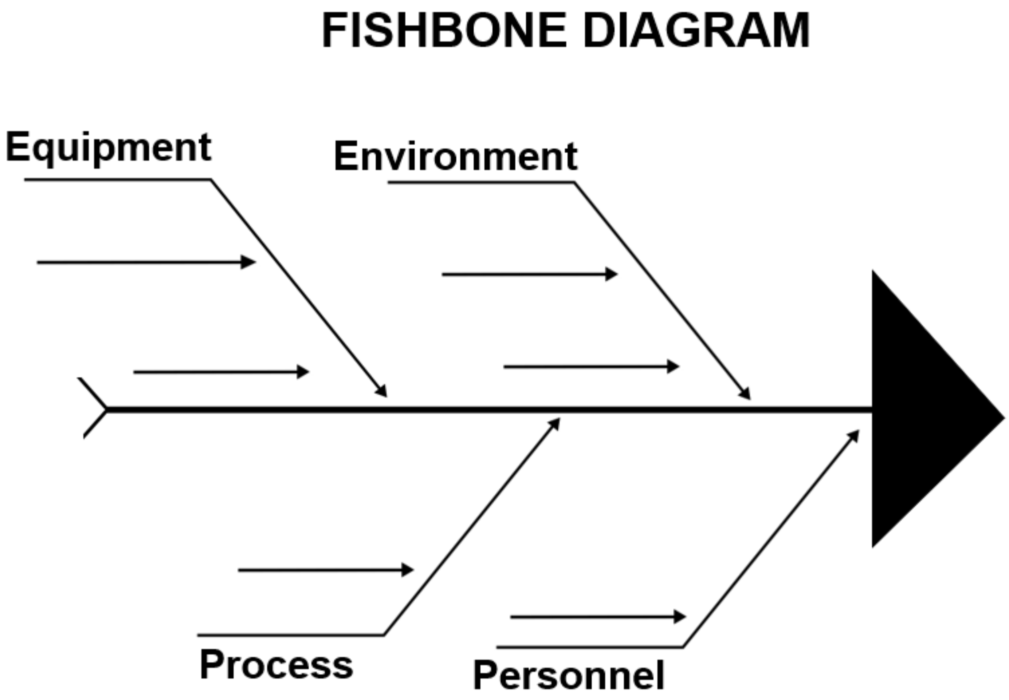
Task 3.1: What are you going to do about it?<br><br>In QI vocab: what is your proposed intervention? Link it to your fishbone diagram.<br><br>This should take you around **10 minutes.**	
Task 4: Pick ONE of financial, social, or environmental sustainability and give ONE reason why your project meets the criteria.<br><br>This should take you around **10 minutes.**	
We want to know how you found your QI funding application process today! Let us know what your 2 take-home messages from today's session were.	
