# Supplementary material for: Using student-staff partnership to teach early years medical students about quality improvement: an evaluation
Source: BMC Med Educ. 2025 Feb 18;25:263. doi: 10.1186/s12909-025-06779-7 (PMC11837700; doi:10.1186/s12909-025-06779-7)
Supplement: Supplementary file 2 — Supplementary Material 2 [file 12909_2025_6779_MOESM2_ESM.pdf]

Worksheet Number: 12

Number of people in your group: 6

Thank you for considering PVB Quality Improvement for your QI funding needs! We want to support YOU to achieve your NHS quality boosting goals. Before we can fund your project, we need to know more about the project you have planned. This easy form should take no more than the hour allocated in your session. To help you do this you have supportive materials on Insendi.

At the end of the session please place your form on the lead tutors' table if you are happy for it to be used as part of the student research project. You will also need to fill in a consent form via Qualtrics. This has been explained at the start of the session and on Insendi.

Well done for submitting your proposal to improve the quality of the service. We appreciate the thought you have put into the proposal and will give it proper consideration!

Task 1 - What problem does your QI project address?

Sell us with a one sentence summary.

This should take you around **10 minutes.**

Patients with English as not their first language may find it difficult to book appointments and access services, online.

Task 2.1: Who does this problem matter to?

That is, who are your stakeholders?

Complete the stakeholder analysis diagram.

Bonus points: tell us how you would contact them (consider other means than surveys)!

This should take you around **15 minutes.**

Patients experiencing digital inequalities

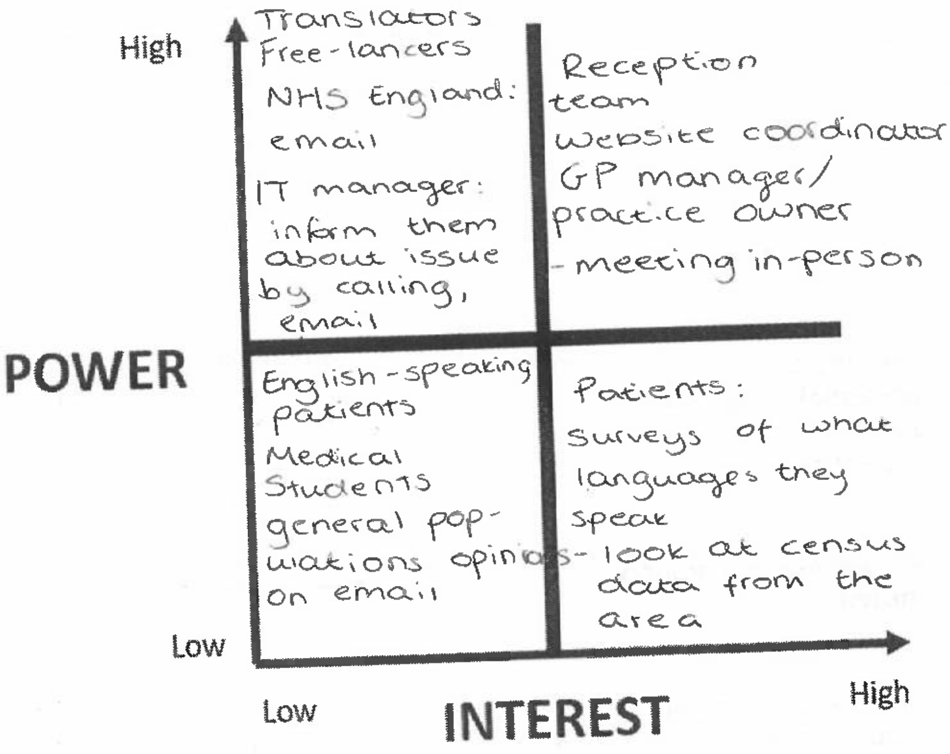

Task 2.2 - What makes the problem, a problem?<br><br>(Hint: Complete the fishbone diagram (aim to put at least two reasons per branch or add your own branches!))<br><br>This should take you around **15 minutes.**	**FISHBONE DIAGRAM** 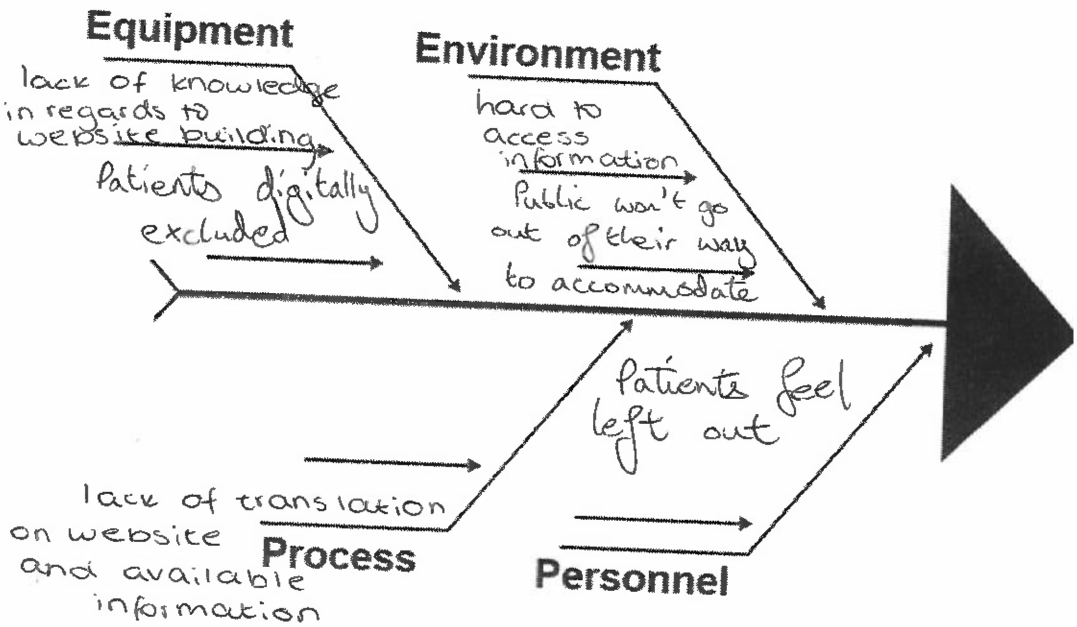
Task 3.1: What are you going to do about it?<br><br>In QI vocab: what is your proposed intervention? Link it to your fishbone diagram.<br><br>This should take you around **10 minutes.**	• Hire IT team to translate local GP pages & texting services to languages which are common in local population. Work with language interpreter.<br>• When logged into website an option to select preferred language and ~~tran~~ automatic translation.<br>• When phone number taken from patients, get them to choose preferred language they want to receive text and notifications in.
Task 4: Pick ONE of financial, social, or ~~environmental~~ sustainability and give ONE reason why your project meets the criteria.<br><br>This should take you around **10 minutes.**	Socially sustainable<br>↳ More people feel included; ↑ accessibility to more patients<br>↳ Makes lives better for those who don't speak English as first language, able to ~~talk about~~ book appts. and investigate symptoms.
We want to know how you found your QI funding application process today! Let us know what your 2 take-home messages from today's session were.	Quality Improvement is a tough process that has many different aspects that you need to consider in order for it to be successful.
